# Effect of Arbuscular Mycorrhizal Fungi (AMF) on photosynthetic characteristics of cotton seedlings under saline-alkali stress

**DOI:** 10.1038/s41598-024-58979-8

**Published:** 2024-04-15

**Authors:** Zicheng Peng, Tayyaba Zulfiqar, Haichang Yang, Ming Wang, Fenghua Zhang

**Affiliations:** Agricultural College, Shihezi University, Shihezi, 832003 Xinjiang People’s Republic of China

**Keywords:** Saline-alkali stress, arbuscular mycorrhizal fungi, Cotton, Growth and photosynthetic traits, Photosynthesis, Plant ecology, Plant physiology, Plant stress responses

## Abstract

The study aimed to find the best Arbuscular Mycorrhizal Fungi (AMF) strain for cotton growth in Xinjiang's salinity and alkali conditions. Cotton (Xinluzao 45) was treated with Funneliformis mosseae (GM), Rhizophagus irregularis (GI), and Claroideoglomus etunicatum (GE) as treatments, while untreated cotton served as the control (CK). Salinity stress was applied post-3-leaf stage in cotton. The study analyzed cotton's reactions to diverse saline-alkali stresses, focusing on nutrient processes and metabolism. By analyzing the growth and photosynthetic characteristics of plants inoculated with Funneliformis mosseae to evaluate its salt tolerance. Saline-alkali stress reduced chlorophyll and hindered photosynthesis, hampering cotton growth. However, AMF inoculation mitigated these effects, enhancing photosynthetic rates, CO_2_ concentration, transpiration, energy use efficiency, and overall cotton growth under similar stress levels. GM and GE treatments yielded similar positive effects. AMF inoculation enhanced cotton plant height and biomass. In GM treatment, cotton exhibited notably higher root length than other treatments, showing superior growth under various conditions. In summary, GM-treated cotton had the highest infection rate, followed by GE-treated cotton, with GI-treated cotton having the lowest rate (GM averaging 0.95). Cotton inoculated with Funneliformis mosseae, Rhizophagus irregularis, and Claroideoglomus etunicatum juvenile showed enhanced chlorophyll and photosynthetic levels, reducing salinity effects. Funneliformis mosseae had the most significant positive impact.

## Introduction

**Figure 1:**
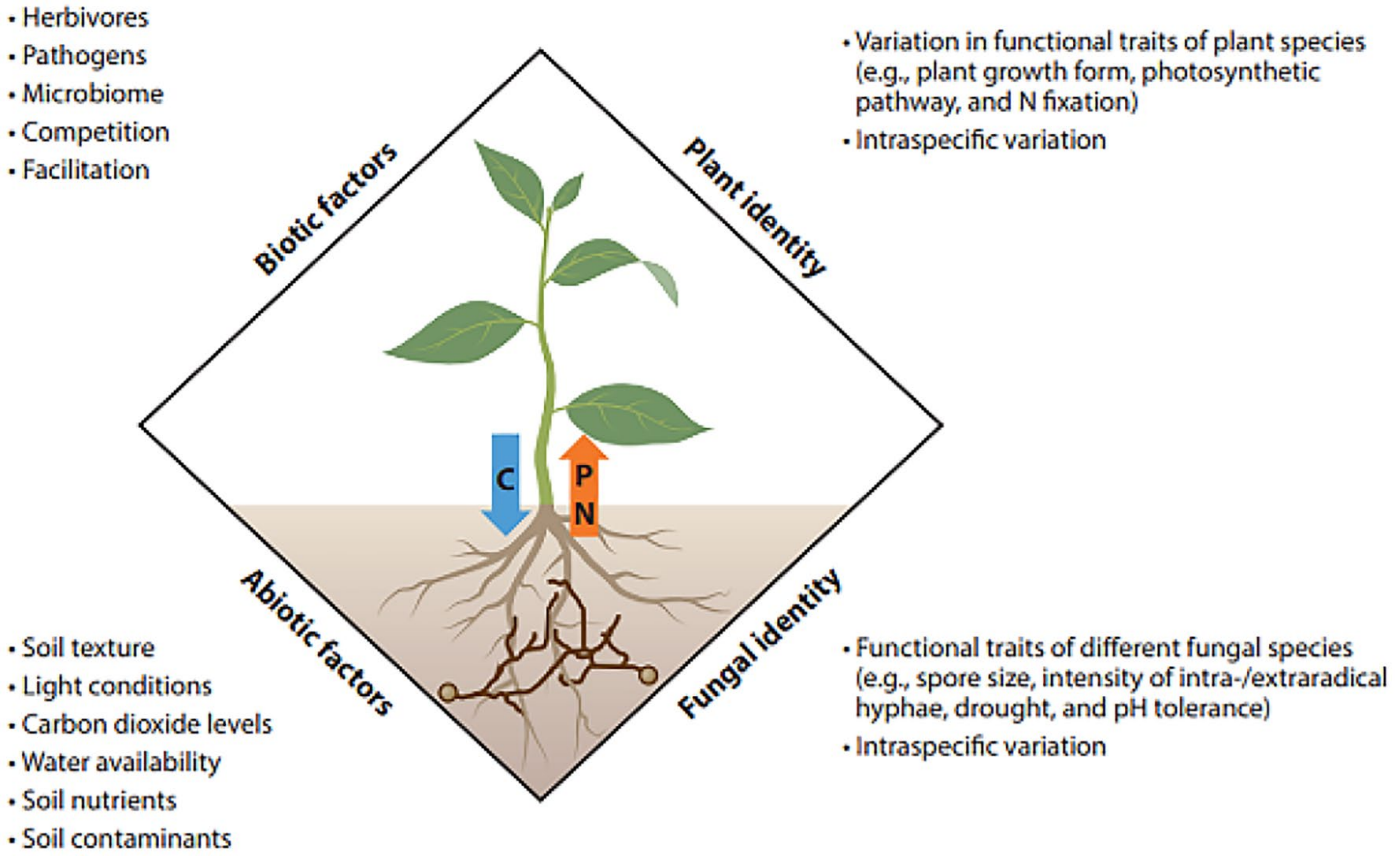
The interaction between plant and arbuscular mycorrhizal fungi (AMF) is influenced by biodiversity and specific environment.

China stands as the leading global producer of cotton, with cotton being a pivotal economic crop in the country. Xinjiang, owing to its favorable climate for cotton cultivation^1^, serves as China's primary hub for high-quality cotton production. Approximately 40% of the nation's total cotton cultivation area and yield are contributed by Xinjiang^2,3^. Despite its prominence, cotton production growth has stagnated due to varying degrees of soil salinization, a challenge particularly prevalent in Xinjiang. China grapples with over 953 million hectares of saline-alkali land, with Xinjiang alone accounting for 22.1% of this area, totaling 2181.4 × 10^4^ hectares^4^. Xinjiang faces severe soil salinization issues, underscoring the urgent need for effective measures to enhance agricultural productivity in the region. Currently, strategies to combat soil salinization primarily focus on water conservation, chemistry, physics, agricultural technology, and biological methods. Among these, biological approaches are widely regarded as the most efficient means of improvement^5^. Biological enhancement primarily involves utilizing salt-tolerant crops and soil microorganisms to absorb and transfer salt, thereby reducing soil salinity. Notably, soil microorganisms can establish symbiotic relationships with crop roots, further aiding in the reduction of salinity levels^6,7^. This review aims to consolidate information on arbuscular mycorrhizal (AM) associations, with a specific focus on their positive impacts on host plants and soil. Initially, we explore the involvement of AM fungi (AMF) in enhancing plant nutrition, promoting growth, and bolstering resistance against both biotic and abiotic stresses. Subsequently, we delve into the indirect role of AMF in fostering soil aggregation and stability. Lastly, we analyze the various interactions between AMF and other soil microorganisms, highlighting the diverse nature of these relationships (Fig. 1).

Arbuscular mycorrhiza (AM) represents a common symbiotic relationship between plant roots and mycorrhizal fungi, prevalent in natural ecosystems. In agricultural settings, it is the predominant type of mycorrhizal association formed by crops^8^. Arbuscular mycorrhizal fungi (AMF) are integral components of terrestrial ecosystems, forming symbiotic relationships with most plant species. Consequently, they hold significant importance within the realm of mycorrhizal fungi. Research has indicated that AMF can influence various physiological processes in plants, facilitating nutrient absorption, increasing aboveground biomass accumulation, and improving light energy utilization efficiency by enhancing photosynthetic pigments and gas exchange parameters^9^. Furthermore, studies have demonstrated the positive impact of AMF combined with biochar on soil pH and nutrient levels in heavy metal-contaminated soil^10^. However, existing research predominantly concentrates on the effects of AMF on plant physiological traits, with limited exploration into the influence of different AMF strains on plant photosynthetic characteristics and soil nutrients under mixed saline-alkali stress^11^.

Therefore, this study employed cotton as the experimental subject to investigate the impact of various AMF strains on chlorophyll content, photosynthesis, plant morphology, root infection rates, soil nutrients, and cotton nutrient levels under saline-alkali stress conditions^12^. The objective was to assess the mitigating effects of AMF on cotton subjected to saline-alkali stress. The study aimed to discern potential disparities in the effects of different AMF inoculations on plant growth and photosynthetic physiology under saline-alkali stress^13^. Additionally, the research sought to identify dominant strains conducive to cotton growth and soil improvement in saline-alkali conditions. These findings provide a theoretical foundation for cotton cultivation and innovative approaches to enhance Xinjiang's saline-alkali land^14^.

## Results

### Various fungi impacted cotton plant shape

The results revealed that the introduction of arbuscular mycorrhizal fungi (AMF) significantly enhanced cotton growth and development, with varying effects observed among different AMF strains. Particularly, cotton growth was optimal under the GM treatment, both in stress-free and stress-induced conditions (Table 1) outlines the impact of different arbuscular mycorrhizal fungi on cotton plant morphology. It is evident from the table that plant height and root length exhibited consistent patterns following AMF inoculation under both non-stress and stress conditions. Plant height notably surpassed other treatments under the GM treatment, displaying a 26.33% increase compared to the control (CK). Moreover, both GI and GE treatments resulted in significantly higher values than CK, with no noteworthy differences between the two. Root length reached its peak under the GM treatment, registering a 20.01% increase over CK.

**Table 1:** Effect of different arbuscular mycorrhizal fungi on cotton plant morphology.

Treatment		Shoot height (cm)	Root length (cm)	Ground fresh weight (g)	Dry weight on the ground (g)	Root fresh weight (g)	Root dry weight (g)
Non-salinity-alkalinity stress	CK_0_	19.59 ± 0.27c	21.09 ± 1.51b	6.42 ± 0.19b	2.29 ± 0.32b	2.25 ± 0.43b	0.43 ± 0.05b
	GM_0_	24.67 ± 0.58a	25.31 ± 1.50a	8.51 ± 0.41a	2.85 ± 0.22a	2.65 ± 0.35ab	0.50 ± 0.07a
	GI_0_	21.33 ± 0.53b	21.32 ± 2.52b	7.85 ± 0.35ab	2.73 ± 0.64ab	2.91 ± 0.53a	0.45 ± 0.04ab
	GE_0_	20.67 ± 0.73b	20.79 ± 2.24b	8.27 ± 0.44a	2.66 ± 0.33ab	2.48 ± 0.28ab	0.46 ± 0.07ab
Salinity-alkalinity stress	CK_S_	16.66 ± 0.57c	20.13 ± 1.26b	6.33 ± 0.34c	2.29 ± 0.22b	2.02 ± 0.12b	0.21 ± 0.06a
	GM_S_	23.42 ± 1.00a	25.52 ± 1.37a	8.68 ± 0.11a	2.81 ± 0.29a	2.45 ± 0.12a	0.27 ± 0.07a
	GI_S_	20.33 ± 0.35b	22.15 ± 2.42b	7.82 ± 0.44b	2.57 ± 0.43ab	2.44 ± 0.15a	0.23 ± 0.06a
	GE_S_	19.46 ± 0.83b	21.79 ± 2.12b	7.80 ± 0.45b	2.65 ± 0.34ab	2.32 ± 0.17a	0.25 ± 0.05a

Different lowercase letters in the same column indicate significant differences among different bacterial treatments at the same salt alkali level (*P* < 0.05).

Under non-stress conditions, GM treatment led to significantly higher aboveground and root dry weights compared to CK, whereas no substantial differences were observed between GI and GE treatments relative to CK. Conversely, under stress conditions, GM treatment exhibited a remarkable 80.74% increase in aboveground dry weight compared to CK and GE treatments. Root dry weight was notably superior under the GM treatment in comparison to other treatments. Furthermore, GM and GI treatments resulted in significantly higher root fresh weights than CK in stress-free conditions, with all inoculation treatments surpassing CK under stress conditions. Aboveground fresh weight was significantly greater under GM and GE treatments compared to CK, with GM treatment outperforming other treatments under saline-alkali stress. In summary, considering cotton morphology, GM outshines the other two strains in enhancing cotton structure and growth.

### Different fungi affected cotton root infection rates

**Figure 2:**
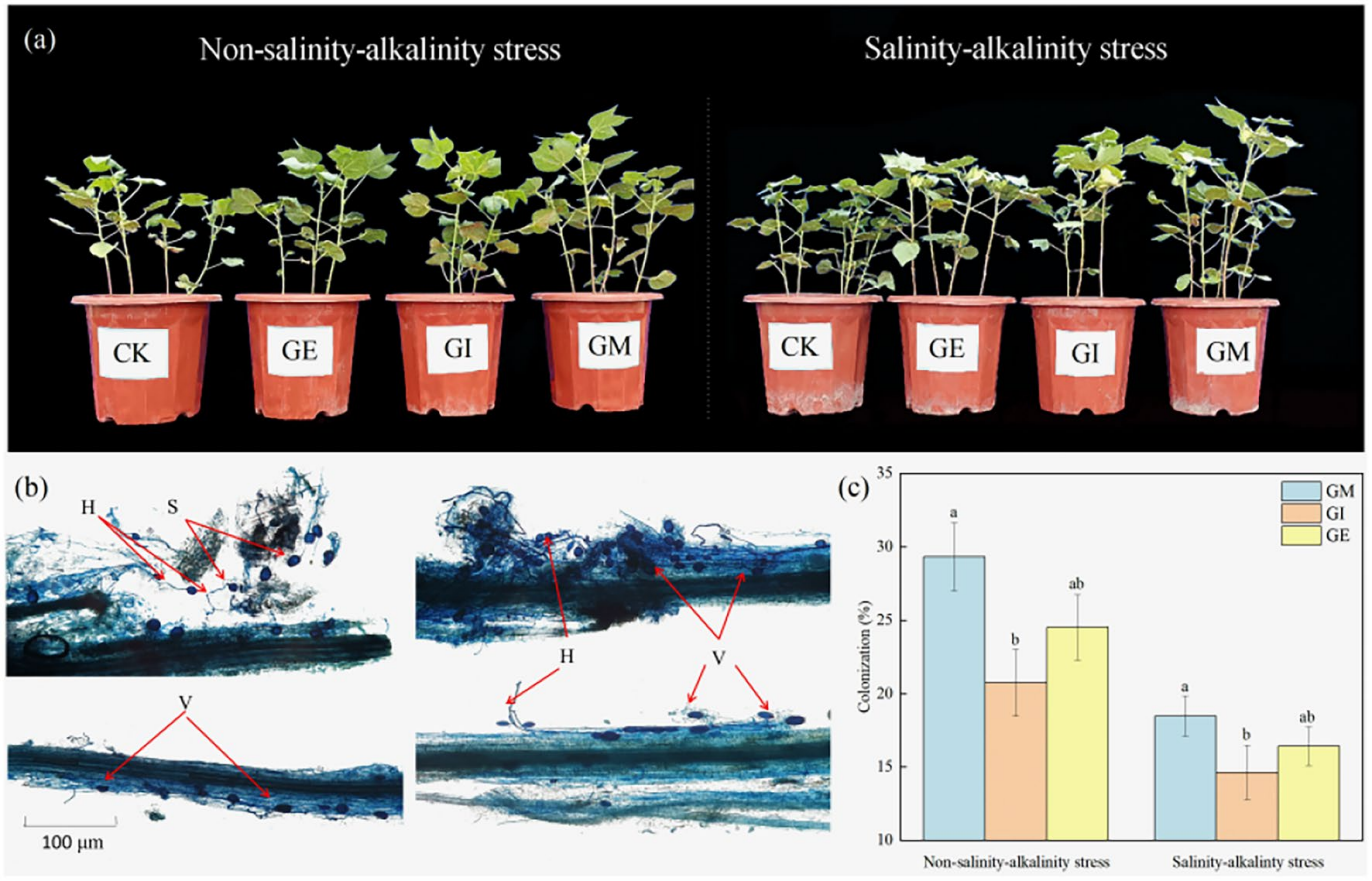
(**a**) Cotton plant morphology after inoculation of different Arbuscular Mycorrhizal Fungi. (**b**) Spores, hyphae and vesicles of cotton root arbuscular mycorrhizal fungi. (**c**) Effect of different arbuscular mycorrhizal fungi on cotton root infection rate. CK (Sterilizing Matrix), GM (Funneliformis mosseae), GI (Rhizophagus irregularis), GE (Claroideoglomus etunicatum), S: Spores, H: Mycelia, V: Vesicles.

The microscopic analysis of cotton root segments depicted well-defined hyphae, vesicles, and spore structures, confirming the presence of arbuscular mycorrhizal fungi (AMF) colonization in cotton roots (Fig. 2). The majority of root segments exhibited AMF infection, with an average infection rate of 36.45% under the inoculation treatment. Examining the impact of different arbuscular mycorrhizal fungi on cotton root infection rates (Fig. 2), it is evident that in the absence of saline-alkali stress, the infection rates followed the order GM > GE > GI, measuring 29.33%, 20.75%, and 24.53%, respectively. Even under saline-alkali stress, GM treatment maintained the highest infection rate, with no significant difference observed compared to GI and GE treatments. The infection rates were ranked as GM > GE > GI, reaching 18.47%, 14.63%, and 16.42%, respectively.

### Different fungi affected cotton leaf chlorophyll levels

**Figure 3:**
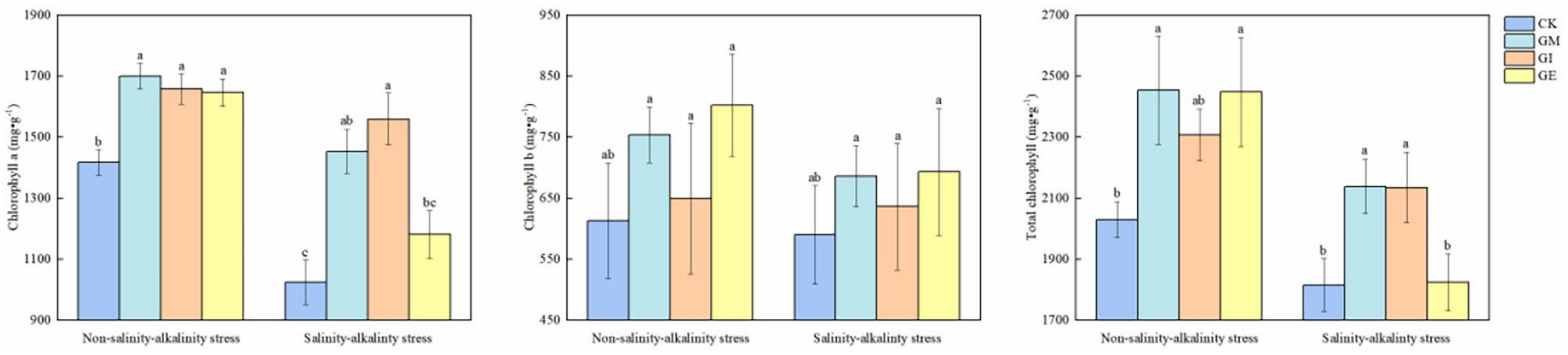
Effect of different arbuscular mycorrhizal fungi on chlorophyll in cotton leaves. Different lowercase letters indicate significant differences between different bacterial treatments at the same salt alkali level (P < 0.05), the different lowercase letters indicate significant differences among treatments (P < 0.05).

The impact of various arbuscular mycorrhizal fungi on cotton leaf chlorophyll levels is presented in Fig. 3. It was observed that the presence of saline-alkali stress led to reduced chlorophyll a, chlorophyll b, and total chlorophyll contents in cotton leaves compared to the non-stress treatment. In non-stress conditions, AMF inoculation significantly elevated chlorophyll content in all treatments when compared to the control (CK). Specifically, GM, GI, and GE treatments exhibited increases of 20.00%, 17.20%, and 16.17%, respectively, in comparison to CK, with no significant differences noted between the inoculation treatments. Under stress conditions, chlorophyll a content in GI and GM treatments significantly surpassed that of CK, with no notable difference between GE and CK. Chlorophyll b content did not show significant differences between treatments with or without saline-alkali stress. Total chlorophyll content in GM and GE treatments significantly exceeded that of CK in unstressed conditions, displaying increases of 20.87% and 20.62% over CK, respectively, with no significant disparity between the inoculation treatments. When subjected to saline-alkali stress, GM and GI treatments exhibited significantly higher total chlorophyll content than CK, marking increments of 17.88% and 17.71%, respectively.

### Various fungi impacted cotton photosynthesis

**Figure 4:**
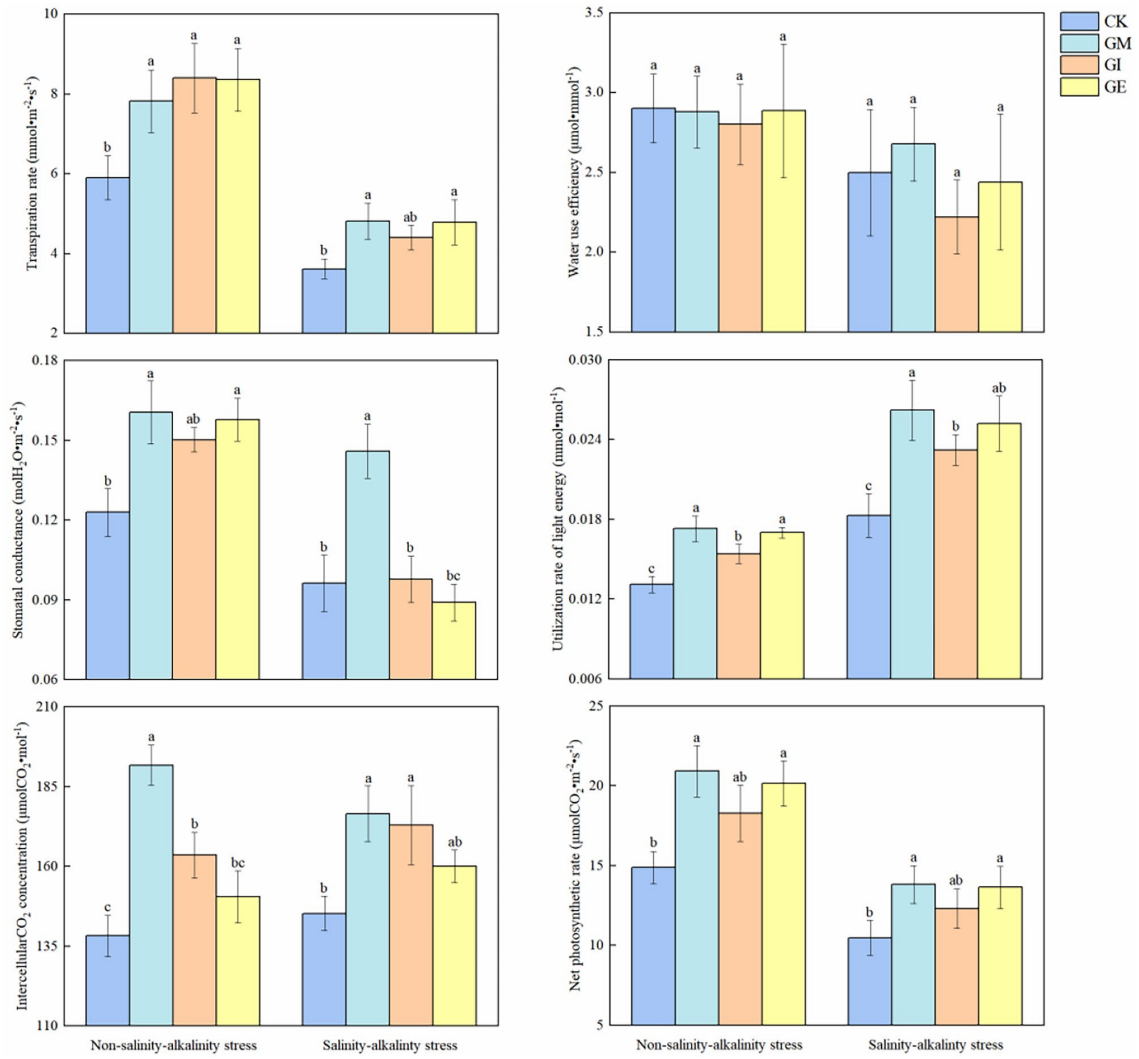
Effect of different arbuscular mycorrhizal fungi on photosynthetic parameters of cotton.

The impact of various arbuscular mycorrhizal fungi on cotton leaf chlorophyll levels is presented in Fig. 4. It was observed that the presence of saline-alkali stress led to reduced chlorophyll a, chlorophyll b, and total chlorophyll contents in cotton leaves compared to the non-stress treatment. In non-stress conditions, AMF inoculation significantly elevated chlorophyll content in all treatments when compared to the control (CK). Specifically, GM, GI, and GE treatments exhibited increases of 20.00%, 17.20%, and 16.17%, respectively, in comparison to CK, with no significant differences noted between the inoculation treatments. Under stress conditions, chlorophyll a content in GI and GM treatments significantly surpassed that of CK, with no notable difference between GE and CK. Chlorophyll b content did not show significant differences between treatments with or without saline-alkali stress. Total chlorophyll content in GM and GE treatments significantly exceeded that of CK in unstressed conditions, displaying increases of 20.87% and 20.62% over CK, respectively, with no significant disparity between the inoculation treatments. When subjected to saline-alkali stress, GM and GI treatments exhibited significantly higher total chlorophyll content than CK, marking increments of 17.88% and 17.71%, respectively.

### AMF boosted cotton photosynthesis in saline-alkali conditions

**Figure 5:**
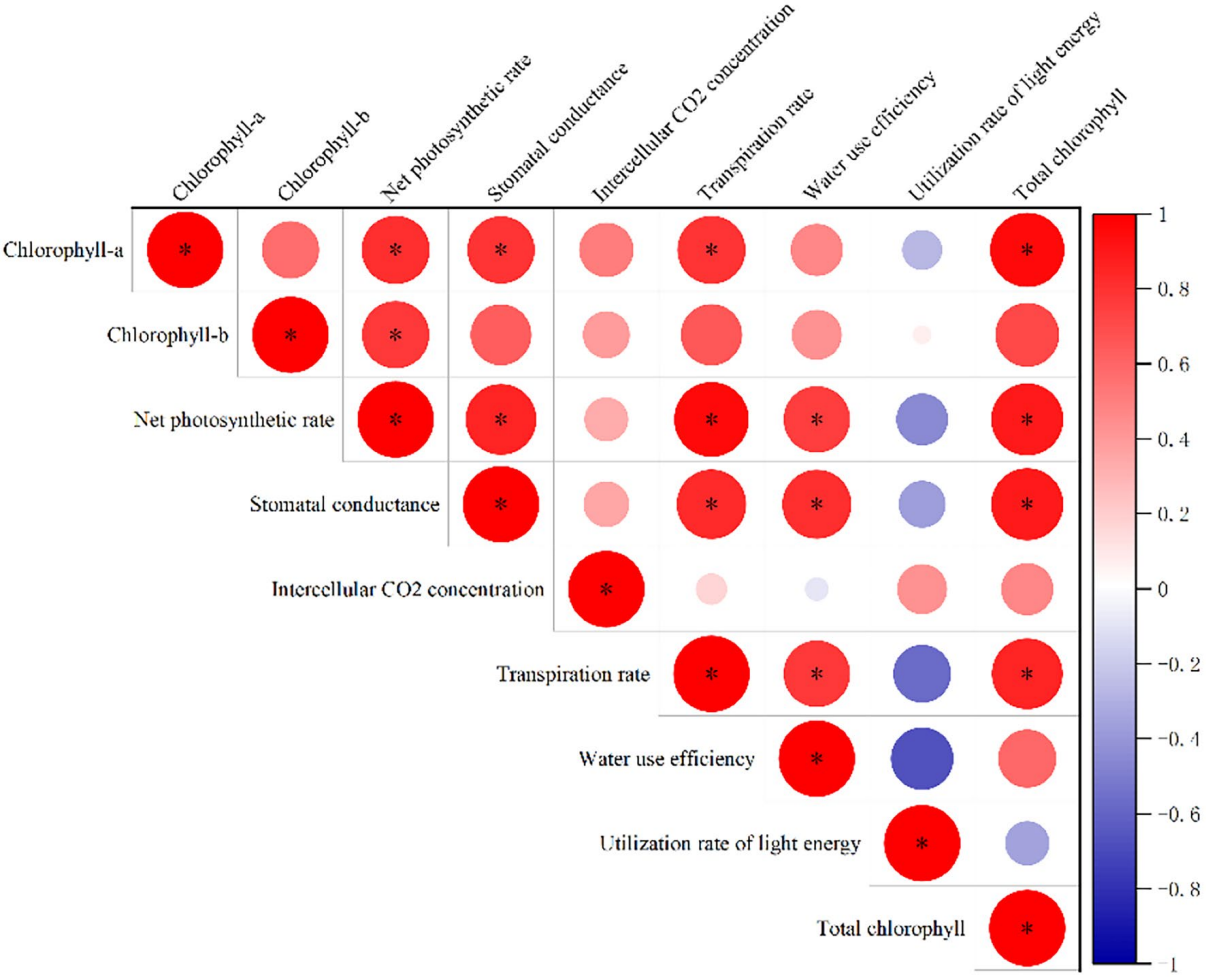
Correlation analysis of indicators under different treatments.

Total chlorophyll was found to have a significant positive correlation with stomatal conductance and a significant positive correlation with net photosynthetic rate, transpiration rate, and light energy utilization rate, according to correlation analysis. The rates of transpiration, light energy use, and stomatal conductance all showed a substantial positive correlation with net photosynthetic rate. Significantly negative correlations were found between stomatal conductance and water use efficiency, and substantial positive correlations were found between it and transpiration rate and light energy utilization rate. Significantly, there was a negative correlation between transpiration rate and water use efficiency, and a positive correlation between transpiration rate and light energy use rate (Fig. 5).

### AMF analysis influenced cotton seedlings light and indices

This experiment chose the chlorophyll a, chlorophyll b, total chlorophyll, net photosynthetic rate, stomatal conductance, intercellular CO_2_ concentration, transpiration rate, water use rate, and light energy utilization rate of each microbial agent treatment in order to thoroughly assess the benefits and drawbacks of each treatment and prevent the bias of single index analysis. The membership function method provided a detailed evaluation of nine indicators. All of the indices are favorable. Each index's average membership function value was sorted (Table 2). The GM treatment had the greatest average membership function value, at 0.95, followed by the GE treatment, which had an average membership function value of 0.76. GI had the lowest average membership function value, at 0.56.

**Table 2:** Membership function analysis of each index.

Treatment	CK		GM		GI		GE	
	Non-salinity-alkalinity stress	Salinity-alkalinity stress	Non-salinity-alkalinity stress	Salinity-alkalinity stress	Non-salinity-alkalinity stress	Salinity-alkalinity stress	Non-salinity-alkalinity stress	Salinity-alkalinity stress
Chlorophyll-a	0.00	0.00	1.00	0.80	0.85	1.00	0.81	0.29
Chlorophyll-b	0.00	0.00	0.74	0.93	0.19	0.45	1.00	1.00
Total chlorophyll	0.00	0.00	1.00	1.00	0.66	0.66	0.99	0.99
Net photosynthetic rate	0.00	0.00	1.00	1.00	0.56	0.55	0.87	0.95
Stomatal conductance	0.00	0.13	1.00	1.00	0.73	0.15	0.93	0.00
Intercellular CO_2_ concentration	0.00	0.00	1.00	1.00	0.47	0.89	0.23	0.47
Transpiration rate	0.00	0.00	0.77	1.00	1.00	0.66	0.98	0.98
Water use efficiency	1.00	0.61	0.77	1.00	0.00	0.00	0.84	0.48
Utilization rate of light energy	0.00	0.00	1.00	1.00	0.55	0.62	0.93	0.87
Average value	0.11	0.08	0.92	0.97	0.56	0.55	0.84	0.67
Mean value in the inoculum	0.10		0.95		0.56		0.76	
Mean value in the inoculum	4		1		3		2	

## Discussion

### Various mycorrhizal fungi affected cotton photosynthesis

Saline-alkali stress significantly impacts cotton seedlings at their early stage, primarily affecting photosynthesis. Chlorophyll, essential for plant photosynthesis, undergoes a reduction in leaves due to the inhibition of necessary element absorption for its synthesis in plant roots under this stress^15^. However, the introduction of arbuscular mycorrhizal fungi (AMF) can enhance root absorption capacity and decrease stomatal limitations, consequently improving leaf photosynthetic capacity^16^. The findings of this research demonstrate that saline-alkali stress leads to decreased levels of chlorophyll a, chlorophyll b, and total chlorophyll in cotton seedlings. Interestingly, inoculation with Funneliformis mosseae and Rhizophagus irregularis significantly elevates total chlorophyll content in cotton seedlings under saline-alkali stress. This indicates the positive impact of AMF inoculation on enhancing photosynthesis in cotton seedlings facing saline-alkali stress. AMF's invasion of cotton roots alleviates salt toxicity, thereby enhancing root nutrient absorption from the surrounding medium. This, in turn, boosts essential element levels for chlorophyll synthesis in aboveground plant parts, facilitating chlorophyll synthesis and metabolism in leaves^17^. Additionally, AMF plays a role in reducing PSII (Photosystem II) sensitivity to photoinhibition by optimizing photon utilization and photosynthetic electron transport. This mechanism helps mitigate the detrimental effects of salinity on host plants^18^.Research indicates that low concentrations of saline-alkali stress can actually enhance chlorophyll synthesis and increase chlorophyll content in leaves. Conversely, high concentrations of saline-alkali stress inhibit growth and significantly decrease chlorophyll levels. This phenomenon is attributed to the presence of proline in cells, which facilitates chlorophyll synthesis. Low levels of saline-alkali stress promote proline accumulation in cells, thereby stimulating chlorophyll production^19^. Furthermore, this study observed a significant increase in the net photosynthetic rate of cotton leaves following inoculation with Funneliformis mosseae and Rhizophagus irregularis. During the development of mycorrhizal roots, AMF growth and reproduction require the utilization of a portion of the host plants' photosynthetic products to form and transport these products^20^.

### Various fungi impacted cotton morphology and infection

Plant morphology provides a straightforward means of understanding a plant's growth status. Root infection serves as a key indicator of the adaptability of arbuscular mycorrhizal fungi (AMF) to plant roots and offers insights into plant growth. In the context of this study, diverse AMF inoculations exerted significant effects on cotton plant height, root length, and overall biomass under saline-alkali stress^21,22^. This suggests that AMF invasion of cotton roots enhances the absorption and utilization of soil nutrients, alters root growth patterns, and fosters cotton growth and development^23^. Research has demonstrated that arbuscular mycorrhizal fungi establish arbuscular structures within host plant roots, facilitating material exchange with the host and forming a mycelial network outside the root. This network extends beyond the reach of the plant's root system, absorbing essential nutrient elements from the surrounding environment. Consequently, the host plant's root system's nutrient absorption range expands, promoting overall plant growth and development^24^. The study revealed variations in cotton root infection rates following inoculation with three distinct AMFs. The root infection rate ranged from 7.65 to 29.33% in response to different inoculation treatments, with Funneliformis mosseae showing the highest infection rate among cotton plants. This variation underscores the distinct responses of different arbuscular mycorrhizal fungi (AMF) to the same host plant, suggesting a mutual selectivity between AMF and host plants. Plants exhibit the ability to choose specific AMF in the soil through their root environment, resulting in different infection rates of various AMF strains within the same host^25^. The infection of AMF in host plants is influenced by both the characteristics of the host plant root environment and the strains of AMF^26^. Studies have revealed that under stressful conditions, AMF inoculation significantly enhances cotton growth and root development, establishing a clear symbiotic relationship with cotton roots. Particularly under stress, cotton root infection rates exceeded 40%. In contrast, cotton plants without AMF exhibited a 0% infection rate, with Funneliformis mosseae treatment resulting in the highest infection rate observed^27^.

## Materials and methods

### Test material

In this study, the experimental strains included Funneliformis mosseae (BGC number: BGC HLJ02A), Rhizophagus irregularis (BGC number: BGC BJ09), and Claroideoglomus etunicatum (BGC number: BGC NM01B), with an average spore density of 26 spores/g. These three arbuscular mycorrhizal fungi (AMF) strains were sourced from the China Arbuscular Mycorrhizal Fungi Germplasm Resource Library at the Beijing Academy of Agriculture and Forestry Sciences. The experiment was conducted in potted plants measuring 15 cm in height and 20 cm in diameter. Xinluzao 45 was the cotton variety used for testing. The soil matrix was collected from the experimental field of Shihezi University, processed by removing stones and fallen leaves, followed by sieving (8 mm) and drying. The soil properties were as follows: pH of 6.03, electrical conductivity (EC) of 0.202 ms cm^−1^, organic matter content of 16.05 g kg^−1^, alkali-hydrolyzable nitrogen content of 51.65 mg kg^−1^, available phosphorus content of 3.375 mg kg^−1^, and available potassium content of 308.17 mg kg^−1^.

### Experimental design

**Figure 6:**
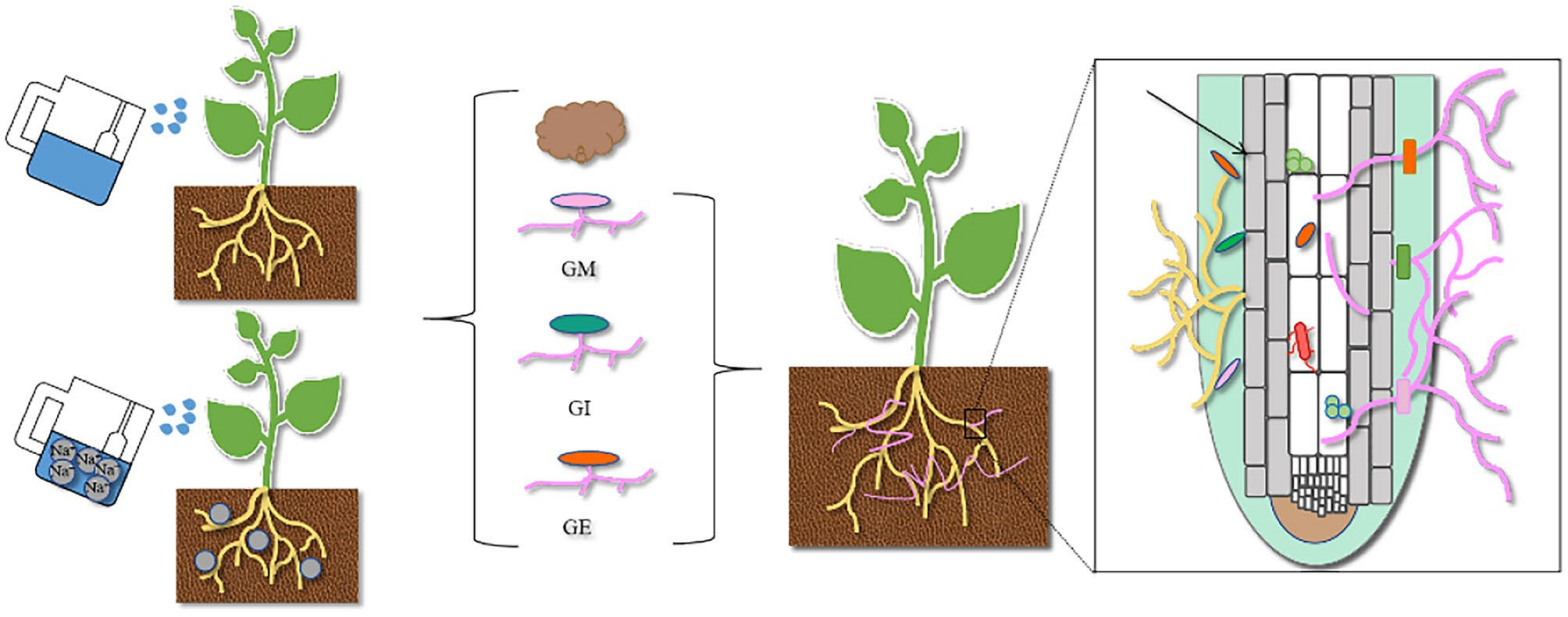
Test treatment diagram. CK (Sterilizing Matrix), GM (Funneliformis mosseae), GI (Rhizophagus irregularis), GE (Claroideoglomus etunicatum).

In the glass greenhouse of Shihezi University, a pot experiment was conducted between April and October 2022. The soil sample was sterilized at 121 °C for 30 min, then cooled to room temperature before adjusting the soil moisture content to 70% of field capacity. Prior to sowing, different treatments were applied, including inoculation with Funneliformis mosseae (GM), Rhizophagus irregularis (GI), Claroideoglomus etunicatum (GE), and a sterilized substrate (CK). During inoculation, the pot was filled with soil up to 3/4 of its capacity, and 20 g of bacterial agent (approximately 520 spores) was evenly spread. The soil was then filled to a total weight of 3.5 kg. In each pot, six cotton plants were sown. Throughout the cotton plant's growth period, 200 ml of water was provided every two days. To induce saline-alkali stress, a mixed saline-alkali solution containing NaCl, Na_2_SO_4_, NaHCO_3_, and Na_2_CO_3_ in a ratio of 12:9:8:1, with a concentration of 100 mmol L^−1^, was applied once a day for 5 consecutive days, following the salt composition of saline-alkali soil in Xinjiang. A control group without saline-alkali stress was maintained. The experiment comprised a total of 8 treatments (Fig. 6), each repeated 4 times, resulting in 32 pots. Later in the experiment, the pots were watered using the weighing method until each pot reached a weight of 3.5 kg. To prevent salt loss, a plastic tray was placed beneath the flowerpot, and any effluent solution was promptly returned to the pot after each irrigation.

### Sample collection

After 4 weeks of stress treatment (seedling stage), the aboveground part was cut off, and the potted soil sample was taken out and placed on craft paper. The roots were gently separated, and the soil near the roots was shaken into a self-sealing bag. The roots were placed in another self-sealing bag. Soil and plant samples were brought back to the laboratory for the determination of each index.Collection of plant material comply with relevant institutional, national, and international guidelines and legislation (Guidelines of the Ministry of Agriculture of the People's Republic of China).

### Determination indicators and methods

#### Chlorophyll

Zhiliang Zhang^28^ proposed a method for chlorophyll determination, which involved grinding 0.5 g of fresh leaves in a mortar with 5 ml of acetone. Subsequently, 5 ml of 80% acetone was added to the mixture. The resulting mixture was then transferred to a centrifuge tube, and after centrifugation, the supernatant was diluted with 80% acetone and subsequently measured.

#### Determination of photosynthetic parameters of leaves

In outdoor conditions with abundant sunlight and calm winds, the LI-6400 photosynthesis instrument and an open gas path system were employed to choose the middle section of the uppermost fully expanded leaf of the cotton plant. This selection allowed for the collection of data on net photosynthetic rate, stomatal conductance, intercellular CO_2_ concentration, and transpiration rate.

#### Plant morphological characteristics

The weight of plants was determined using a weighing method to measure both fresh and dry weights. Additionally, the height and root length of cotton plants were measured using appropriate measurement techniques.

#### Root staining and AMF colonization quantification

Two random cotton roots were chosen, washed with water, and cut into 1 cm segments. These segments underwent a 10% KOH treatment for transparency, followed by a 90 °C water bath for 30 min. After washing, they were acidified with 5% lactic acid and then stained with trypan blue. Under a microscope, each root segment was assessed on a scale of 0, 10, 20, 30, up to 100, with 10 as the initial level. The mycorrhizal infection rate was calculated using the following formula^29^:

1 $$ \% {\text{of root colonization}} = \frac{{{\text{No}}{.}\,\,{\text{of root bits showing colonization}}}}{{\text{Total number of root bits observed}}} \times 100 $$

### Statistical analysis

The experimental data were plotted and statistically analyzed by Microsoft Excel 2021 and SPSS 19.0. Duncan Method for multiple comparisons of differences between treatments. The membership function evaluation method is used to evaluate the best treatment^30^. The specific formula is:

2 $$ UX\left( + \right) = \frac{{\left( {Xij - Ximin} \right)}}{Ximax - Ximin} $$

3 $$UX\left(-\right)=1-UX\left(+\right)$$

## Data Availability

The authors declare that all data supporting the findings of this study are available within the paper.
